# Supplementary material for: Iron Limitation Restores Autophagy and Increases Lifespan in the Yeast Model of Niemann–Pick Type C1
Source: Int J Mol Sci. 2023 Mar 25;24(7):6221. doi: 10.3390/ijms24076221 (PMC10094029; doi:10.3390/ijms24076221)
Supplement: Supplementary file 1 [file ijms-24-06221-s001.zip › Supplementary figures.pdf]

Supplementary figures

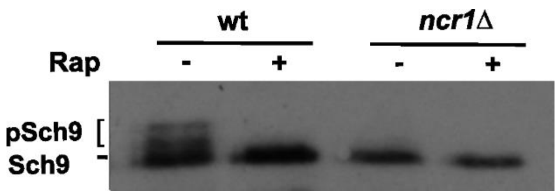

Figure S1. TORC1-mediated Sch9 phosphorylation decreases in *ncr1*Δ cells. Wild type (wt) and *ncr1*Δ cells transformed with pRS416-*SCH9-5HA* plasmid were grown to exponential phase in SC medium and protein extracts treated with NTCB (2-nitro-5-thiocyanatobenzoic acid) were analyzed by immunoblotting using anti-HA antibody. A representative experiment is shown. Cells were treated with rapamycin (Rap) or DMSO as control.

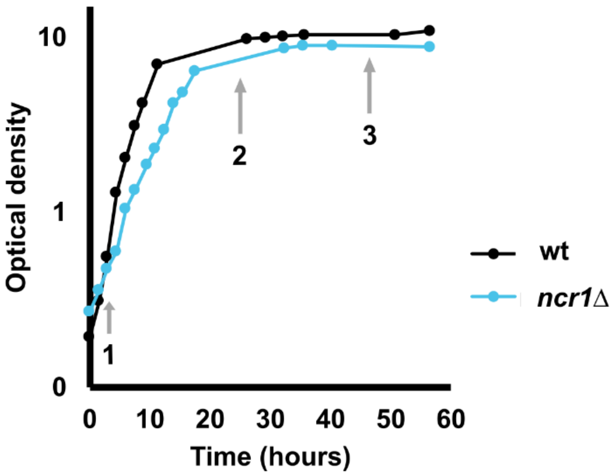

Figure S2. Wild type (wt) and *ncr1*Δ cells were grown in SC media and samples were taken to monitor the OD_{600nm} overtime. Data are the mean values of three independent experiments. Arrows represent the time points where samples were taken for experiments at exponential phase (1) and post-diauxic shift (PDS) phase - 24 h (2) or 48h (3) after exponential phase.

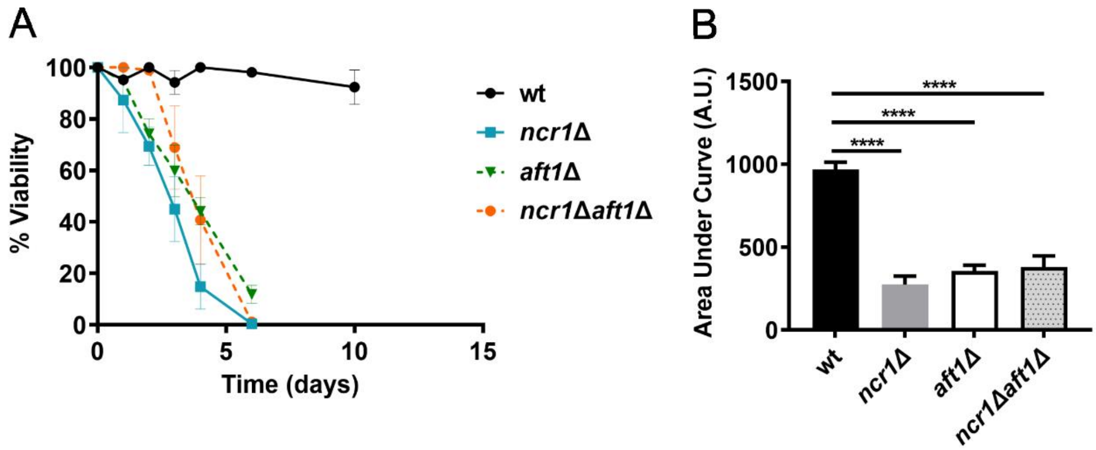

Figure S3. *AFT1* deletion does not have any impact on the chronological lifespan of *ncr1*Δ cells. (A) Wild type (wt), *ncr1*Δ, *aft1*Δ and *ncr1*Δ*aft1*Δ cells were grown to post-diauxic shift (PDS) phase in SC medium and maintained in this medium overtime. Cellular viability was expressed as the percentage of the colony-forming units in relation to day 0. Data are the mean ± SEM of at least three independent experiments. (B) The area under each lifespan curve was computed using GraphPad Prism 8 in arbitrary units (A.U.). Data are the mean ± SEM. ****, p ≤ 0.0001 (one-way ANOVA).

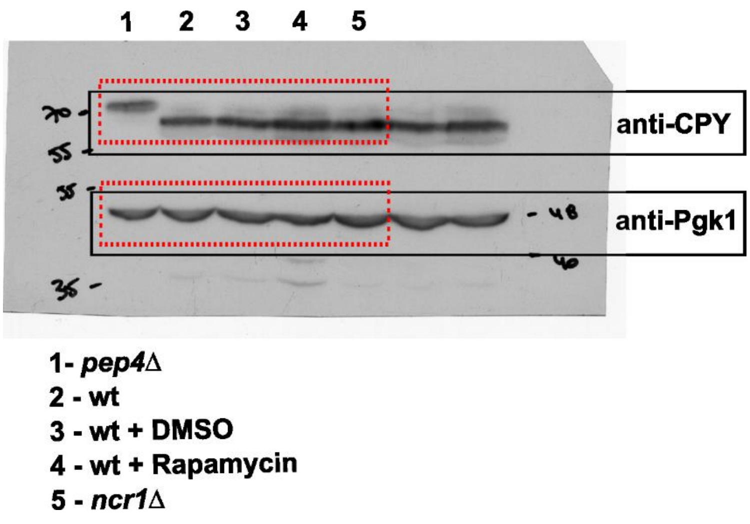

Figure S4. Uncropped and unadjusted full original image of western blot displayed in Figure 3B. Red rectangles are used to highlight where the bands were taken from.

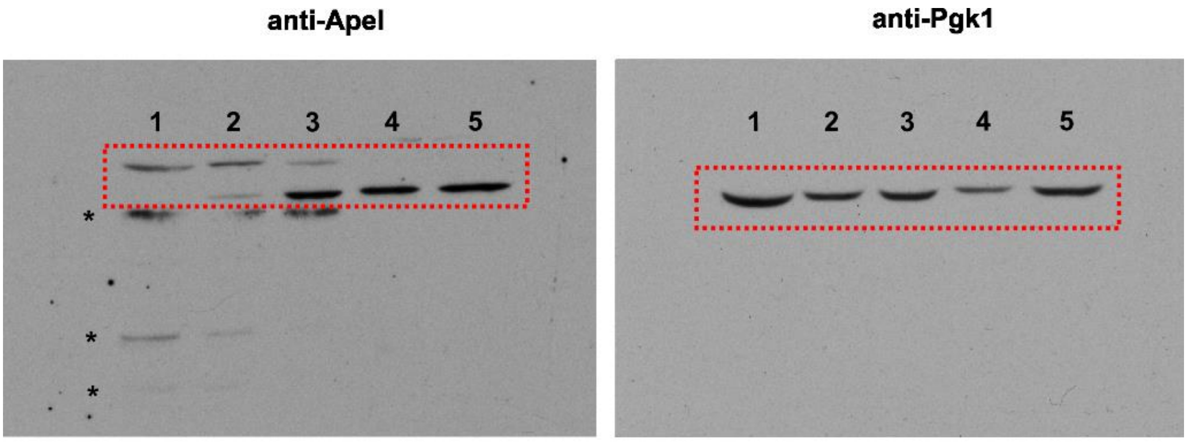

*** - unspecific bands**

1- *pep4*Δ
2- wt exponential phase
3 - *ncr1*Δ exponential phase
4 - wt post-diauxic shift phase
5 - *ncr1*Δ post-diauxic shift phase

Figure S5. Uncropped and unadjusted full original image of western blot displayed in Figure 3C. Red rectangles are used to highlight where the bands were taken from.

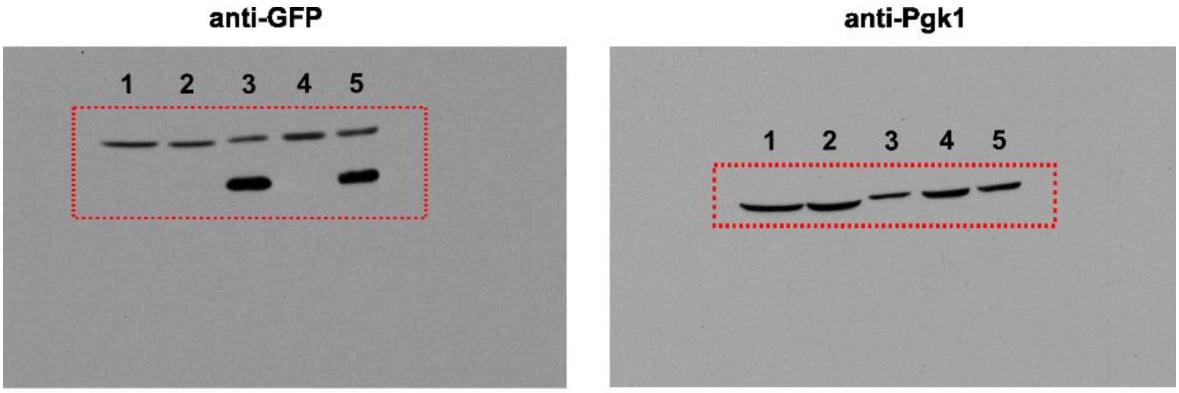

1 - wt exponential phase
2 - *ncr1*Δ exponential phase
3 - wt post-diauxic shift phase
4 - *ncr1*Δ post-diauxic shift phase
5 - wt exponential phase + Rapamycin

Figure S6. Uncropped and unadjusted full original image of western blot displayed in Figure 4A. Red rectangles are used to highlight where the bands were taken from.

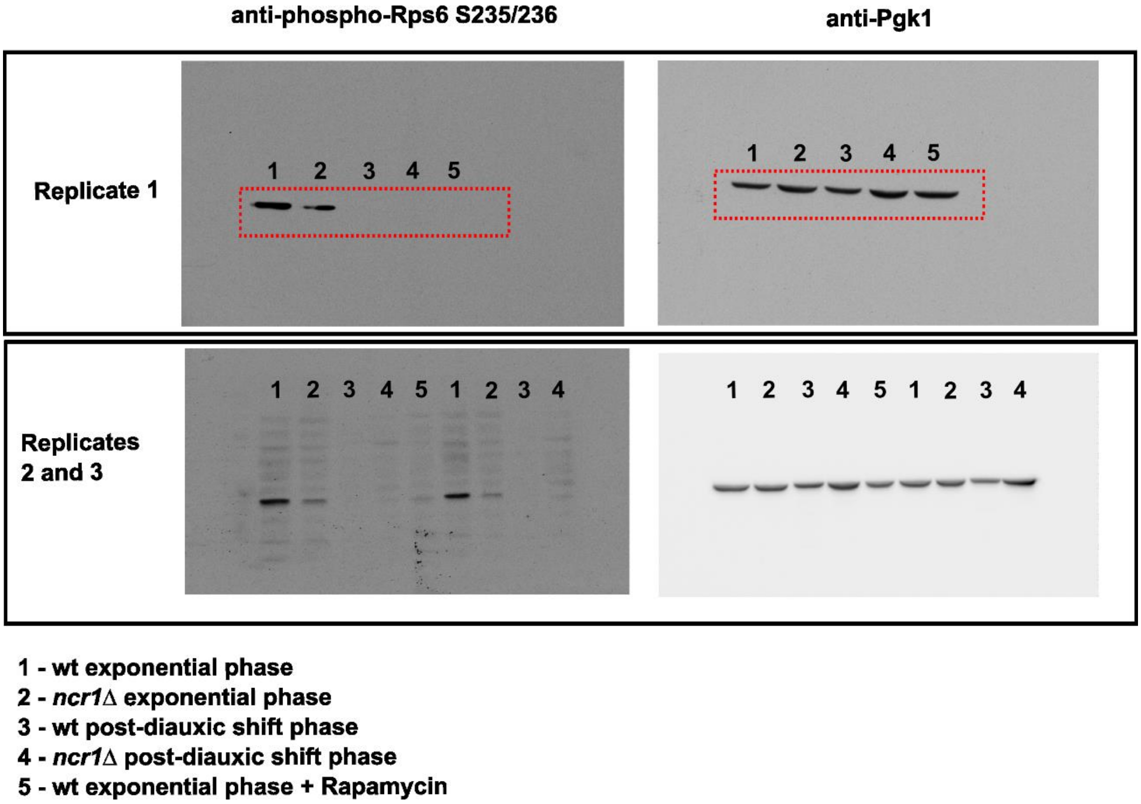

Figure S7. Uncropped and unadjusted full original images of western blots used for data quantification displayed in Figure 4C. Red rectangles are used to highlight the original image of the blots displayed in Figure 4B.

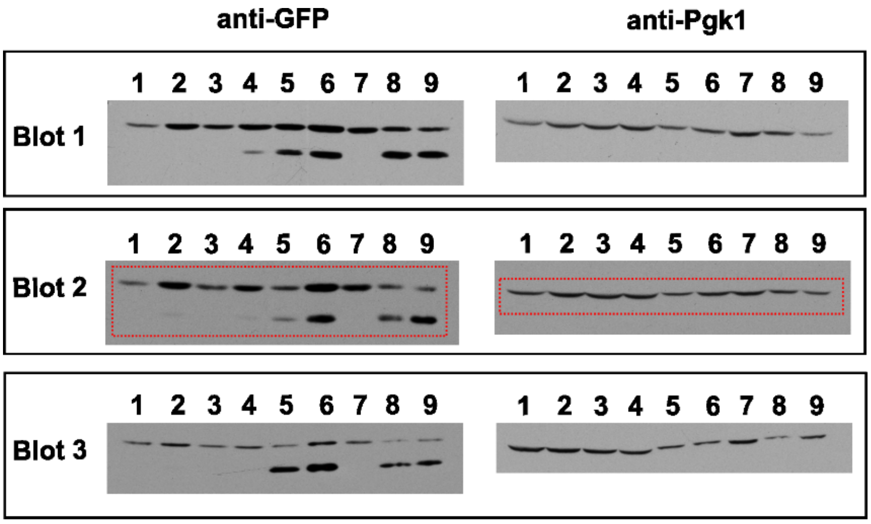

1 - wt exponential phase
2 - wt + BPS exponential phase
3 - *ncr1*Δ exponential phase
4 - *ncr1*Δ + BPS exponential phase
5 - wt post-diauxic shift phase
6 - wt + BPS post-diauxic shift phase
7 - *ncr1*Δ post-diauxic shift phase
8 - *ncr1*Δ + BPS post-diauxic shift phase
9 - wt exponential phase + Rapamycin

Figure S8. Uncropped and unadjusted full original images of western blots used for data quantification displayed in Figures 6G-H. Red rectangles are used to highlight the original image of the blots displayed in Figure 6F.
